# CARE MANAGEMENT CHALLENGES AND PROGNOSTIC TOOLS FOR HEART FAILURE PATIENTS IN HOSPICE

**DOI:** 10.1093/geroni/igz038.2912

**Published:** 2019-11-08

**Authors:** Ruth Masterson Creber, Lizeyka Jordan, Dawon Baik, David Russell

**Affiliations:** 1 Weill Cornell Medical College, New York, New York, United States; 2 Visiting Nurse Service of New York, New York, New York, United States; 3 Columbia University School of Nursing, New York, New York, United States; 4 Appalachian State University, Boone, North Carolina, United States

## Abstract

Heart failure (HF) patients enroll in hospice at lower rates despite their worse prognosis. This multi-method study explores the characteristics and challenges associated with caring for HF patients. Data from qualitative interviews with hospice providers (n=32) and quantitative records (N=1,114) were used to identify care management issues and prognostic tools. Hospice providers described HF patients unique and often unpredictable symptomatology, their limited understanding and discordant hospice expectations, and difficulties managing symptoms at home. Providers also highlighted HF patients use of assistive medical devices and complex medication regimens. Palliative Performance Scale (PPSv2) scores at hospice enrollment were found to be strongly associated with hospice survival (AUC: 7 days=0.80; 14 days=0.77) and live discharge risk (PPSv2 50-70% AOR=5.68 [CI=3.66-8.79]). Findings underscore the need for specially-tailored trainings and protocols for providers to prevent unplanned discharges and support HF patients at end-of-life.

